# Microsatellite Instability and Life Style Factors in Sporadic Colorectal Cancer

**DOI:** 10.31557/APJCP.2020.21.5.1471

**Published:** 2020-05

**Authors:** Nora Atef, Nelly Alieldin, Ghada Sherif, Iman Loay, Ahmed Mostafa Mahmoud, Ghada Mohamed

**Affiliations:** 1 *Biostatistics and Cancer Epidemiology department, National Cancer Institute (NCI), Cairo University, Egypt. *; 2 *Pathology Department, NCI, Cairo University, Egypt. *; 3 *Surgical Oncology department , NCI, Cairo University, Egypt. *

**Keywords:** MSI, CRC, BMI, physical activity, Egypt

## Abstract

**Background:**

Colorectal cancer (CRC) in Egypt is a relatively high young onset disease. As a form of heterogeneous cancer, there is interplay between genetic and environmental factors. We aimed at probing the association of life style factors and Microsatellite Instability (MSI) status that could provide more insights on carcinogenic process of CRC.

**Methods:**

One hundred incident sporadic CRC patients were involved. Information on risk factors of CRC was obtained and microsatellite instability status was predicted through evaluation of MMR protein expression via immunohistochemistry (IHC).

**Results:**

Median age was 47.50 years, females represented 54.0% and 36% of patients were Microsatellite Instability High (MSI-H). Most patients with right sided colon cancer (78.3%) were MSI-H while mostly stable or low MSS/MSI-L for left-sided colon and rectum (78.6%, 74.3% respectively, p<0.001). Patients with low physical activity had higher risk of MSS/MSI-L than those with moderate or high activity p =0.026. Patients with BMI greater than 30 Kg/m^2^ had higher MSS/MSI-L (75.5%) than those with BMI between 25-30 Kg/m^2^ (60.6%) and those with normal BMI <25 (38.9%), p for trend = 0.006. On subgroup analyses, the association of high BMI with MSS/MSI-L was only shown in patients younger than 40 years, females, stage III, non-mucin secreting adenocarcinoma and a significant interaction with physical activity.

**Conclusion:**

In Conclusion, the present study confirms the increased risk of MSS/MSI-L with increased BMI and speculates this association to be modified by patient’s life style and tumor characteristics. Further research is needed to validate present results.

## Introduction

Colorectal cancer (CRC) is one of the leading causes of mortality and morbidity in the world (Favoriti et al., 2016). In Egypt, CRC ranked the 6th, representing about 4% of total cancers in both sexes (Zeeneldin et al., 2012). CRC is a heterogeneous disease that can be classified into distinct subtypes with different underlying molecular alterations, such as microsatellite instability (MSI), chromosomal instability, and the CpG-island methylator phenotype (CIMP) (Jass, 2007). MSI is a kind of genomic instability where mutations occur in nucleotide repeat sequences throughout the genome. These repeat sequences are known as microsatellites, and the discrepancy between these sequences in tumor and germline cells are known as microsatellite instability (Boland and Goel, 2010). MSI arises from defects in DNA mismatch repair (MMR) system (Vilar and Gruber, 2010).

Many risk factors are known in CRC of which two modifiable and interrelated risk factors, excess body weight and physical inactivity, are reported to account for about a fourth to a third of colorectal cancers (Lee et al., 2007).

High body mass index (BMI) is an established risk factor for colorectal cancer (WCRF, 2007; Renehan et al., 2008; Ning et al., 2010; Ning et al., 2013); however, associations are usually stronger for men than women and for colon than rectal cancers (Campbell et al., 2007; Renehan etal., 2008; Ning etal., 2010; Ning et al., 2013). Emerging data suggestthat the BMI-colorectal cancer association differs by microsatellite instability (MSI) status (Slattery et al., 2000; Satia et al., 2005; Campbell etal., 2010; Hughes et al., 2012; Sinicrope et al., 2012), with stronger associations typically shown for MS-stable than MSI-high tumors.

When BMI was evaluated in the peri or post-diagnosis period, generally null or only modest associations were shown (Sinicrope et al., 2012; Campbell et al., 2012). In contrast, when pre-diagnosis BMI was evaluated, studies typically showed higher risks of all-cause and colorectal-cancer-specific mortality with high BMI (Prizment et al., 2010; Campbell et al., 2012; Parkin et al., 2014, Fedirko et al., 2014).

Many studies have investigated associations of risk factors and colorectal cancer by anatomic subsite of the tumor (e.g., colon/rectum, proximal/distal) (Botteri et al., 2008; Harriss et al., 2009; Ning etal., 2010), which may partly reflect associations with different molecular types of carcinogenesis (Renehan et al., 2008). Discrimination of CRC by molecular subtypes could provide clearer distinction of colorectal cancers and their associations with risk factors than tumor subsite alone (Iacopetta, 2002; Yamauchi et al., 2012). Knowledge and understanding of associations of risk factors and MSI subtypes of CRC could contribute to better understanding of the interaction of risk factors and carcinogenic processes. 

The aim of this study was to show frequency of MSI in Egyptian CRC patients and whether there is an association between MSI and risk factors of CRC and if present could it be modified by BMI.

## Materials and Methods

*Study design and study population*

Current study is cross sectional in design. Incident sporadic CRC diagnosed at NCI, Cairo University in the period from January 2017 to January 2018 were included. 

Criteria of inclusion was all ages, both gender, newly diagnosed, sporadic, histologically proved primary colorectal cancer, mental ability to complete and willing to participate. Prevalent cases, those with hereditary colorectal cancer syndromes (FAP, Crohn’s disease and HNPCC) who fulfill Revised Bethesda Guidelines, or advanced stage (stage IV) were not included. Out of 101 patients, one hundred agreed to participate (99%). Sporadic colorectal cancer is the most common type of CRC and includes approximately 75 % of cases that display no apparent evidence of having inheritance of disorder. Sporadic colorectal cancer is common among elder people, probably because of environmental factors, dietary, and aging (Arvelo et al., 2015).

*Data collection*

Personal interviews were conducted during hospitalization, before or just after surgery. Standardized questionnaire with detailed medical and family history, as well as socio-demographic and lifestyle factors was used. The index date was the date of diagnosis. Information on weight before diagnosis and current height were obtained from self-reports during the interview, to calculate BMI of the patients (kg/m^2^). Self-reported weight was shown to be highly accurate compared to standardized measurements (Rimm et al.,1990), weight at least 2 years before the index year was used (e.g., for a person age 65 years we used weight at age 63). This measure of body weight was preferred over current weight because cancer-related symptoms before and after diagnosis or therapy of colorectal cancer can cause weight changes. Detailed data about physical activity at work, leisure time and walking were collected during the interview by the short form of International Physical Activity Questionnaire (IPAQ) (Booth et al., 2000).

*Collection and immunostaining of tumor samples*

Immunohistochemistry (IHC) is now considered a validated method in predicting MSI in colorectal cancer with high sensitivity (93%) and nearly perfect specificity in predicting MSI in CRC as determined by PCR (Lindor et al., 2002; Shia, 2015), but less certain in other cancer types. For seleting CRC cases, diagnosis was confirmed based on examining the Hematoxylin and Eosin (HE) stained sections under the light microscopy. For IHC staining, 4 sections were cut for each CRC case at a thickness of 4 μm from the paraffin-embedded tissues. Sections were then placed onto X-tra adhesive slides. IHC staining was performed using BenchMark XT (Ventana) autostainer according to the protocol instruction. Birefly, deparaffinization followed by cell conditioning for 64 minutes, then antigen retrieval using reaction buffer (PH 7.4-7.8) was carried out. Next, ready-to-use primary monoclonal antibodies were applied under specific incubation temperature and time.

For assessment of mismatch repair (MMR) proteins (MLH1, PMS2, MSI-H2, and MSI-H6), primary antibodies used as follows: VENTANA anti-MLH1 (M1) Mouse Monoclonal Primary Antibody, cat no. 790-5091, VENTANA anti-PMS2 (A16-4) Mouse , cat no. 790-5094, VENTANA anti-MSI-H2 (G219-1129) Mouse Monoclonal Primary Antibody, cat no. 790-5093, VENTANA anti-MSI-H6 (SP93) Rabbit Monoclonal Primary Antibody, cat no. 790-5092.

The reaction is visualized by using DAB (Diaminobenzidine) chromogen as a coloring agent andHematoxylin as a counterstain. Normal colonic mucosa was used as positive control.

*Microsatellite instability analysis*

**Figure 1:**
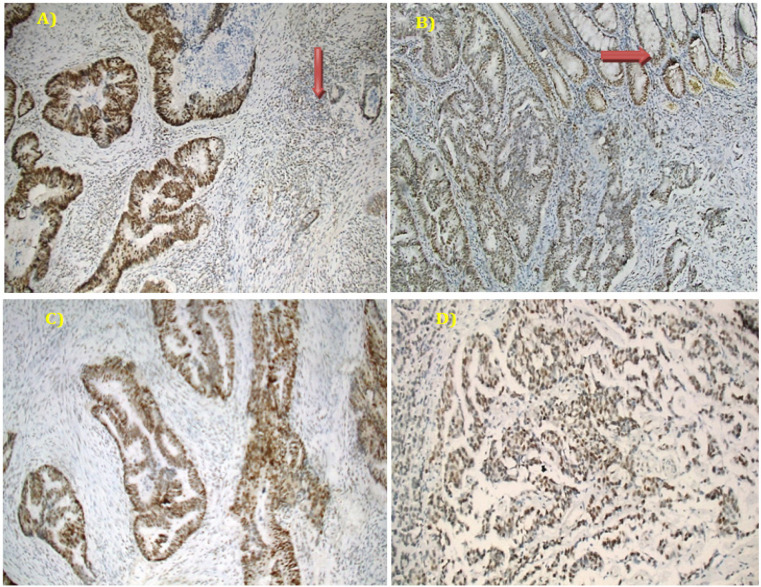
A Representative Example of pMMR (MSS) Colorectal Carcinoma, shows (A) Positive MLH-1 in tumor cells as well as the internal positive control [lymphocytes with red arrow] (Mag. 100X). (B) PMS2 is positive in tumor cells as well as normal colonic mucosa [red arrow] (Mag. 100X). (C) Positive MSH6 in tumor cells (Mag. 100X). (D) Positive MSH2 in tumor cells (Mag. 100X).

**Figure 2:**
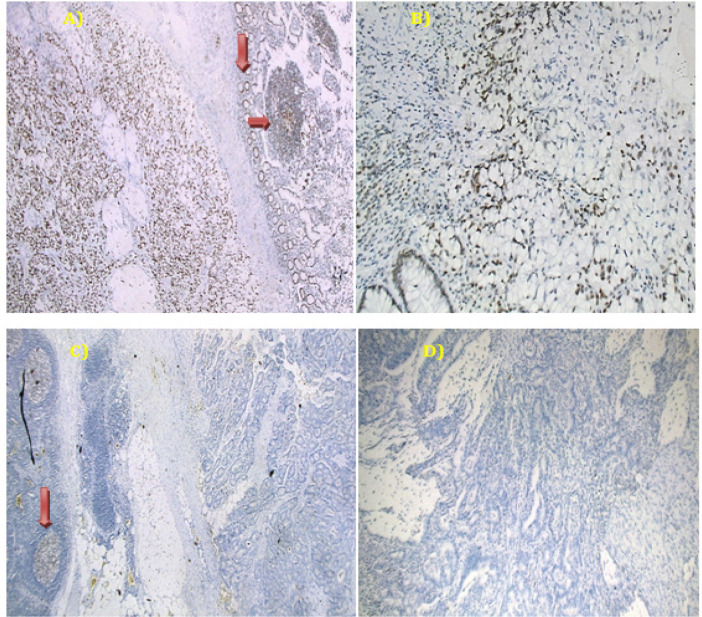
A Representative Example of dMMR (MSI-H) in a Case of Adenocarcinoma with Signet Ring Differentiation. (A) Positive MLH-1 in tumor cells with positive internal control [normal colonic epithelium and lymphoid follicles, red arrows] (Mag. 50X). (B) Positive PMS2 in tumor cells (Mag. 100X). (C) Loss of MSH2 in tumor cells, while positive internal control (lymphoid follicles, red arrow) (Mag. 50X) and (D) loss of MSH6 in tumor cells (Mag 100X).

Cases were categorized into positive (unequivocal positive nuclear staining within tumor cells) and negative (complete absence of nuclear staining within tumor cells with concurrent internal positive controls) (Hughes et al., 2012). Normal colonic mucosa, stromal fibroblasts, lymphocytes or germinal centers of lymphoid follicles were used as positive internal control) (Figures 1 and 2). Tumor displaying loss of at least one mismatch repair protein (MMR protein) can be collectively referred to as MMR-deficient (dMMR) and are considered to be (MSI-H), whereas those with intact MMR proteins can be classified as MMR-proficient (pMMR). pMMR are expected to be microsatellite stable (MSS) or MSI-low (MSI-L) (Kawakami et al., 2015).

*Sample size*

Based on a previous study by Soliman et al., (2001) who found that, frequency of MSI-H tumors in the Egyptian group was 37.0%, while Vilar and Gruber, 2010 reported that prevalence of MSI-H ranged from 15-20%. We assumed a frequency of MSI-H to be 30% and calculated the required sample size to be 81 CRC patients which was sufficient for estimating the expected proportion with 10% absolute precision (0.25 – 0.35) with 95% confidence interval. We increased the number to 100 to allow for non-compliance of patients. Using the above criteria, 36% showed negative expression of at least one of the MMR proteins and were predicted to be MSI-H (Figure 2), while 64% showed retained expression of all MMR proteins and predicted to be MSS/MSI-L (Figure 1).

*Statistical analysis*

Data was analyzed using IBM SPSS advanced statistics (Statistical Package for Social Sciences), version 24 (SPSS Inc., Chicago, IL). Numerical data was described as median and range, while qualitative data was described as number and percentage. Man Whitney test used to compare non-normally distributed numerical variables. Chi-square or Fisher exact tests as appropriate were used for comparing categorical data and calculating risk. Interaction was tested using Breslow Day test. All tests were two tailed and p value set significant at 0.05.

## Results

*Relation of MSI to patient characteristics and life-style*

Out of 100 incident CRC patients, 36% showed loss of the expression of at least one MMR protein (dMMR/MSI-H) and 64% showed preserved expression of all MMR proteins [pMMR /MSS and MSI-L], Figures 1 and 2. The median age was 47.50 with a range of (19- 86) years and females represented 54.0%. Patients aged more than 40 years were found to have slightly higher percent of MSS/MSI-L than those ≤40 years and female patients compared to males without a statistical significance. School education measured in years, family history of CRC, being hypertensive or diabetic did not show a significant relation with MSI status. Most Patients with right sided colon cancer (78.3%) were MSI-H while it was nearly the reverse for left-sided colon and rectum (78.6% and 74.3% MSS/MSI-L respectively), p value <0.001. Whether the tumor was mucin or non-mucin secreting adenocarcinoma, it does not affect MSI status. No significant relation was found between tumor grade, lymph node infiltration or TNM stage and MSI status (Table1).

Former smokers tend to have a higher percent of MSS/MSI-L than current smokers without significance. History of alcohol consumption or use of oral contraceptives pills in females were not found to be related to MSI. Neither consumption of diet with high fiber content (vegetable and fruits) nor fast food diet or processed meat was found to be associated with MSI status. Patients with low physical activity (measured as <600 MET minutes /week) had higher percentage of MSS/MSI-L than those with moderate or high physical activity (p value=0.026). Patients with pre-diagnosis BMI greater than 30Kg/m^2^ had higher MSS/MSI-L (75.5%) than those with BMI between 25-30 (60.6%) and those with normal BMI <25 (38.9%), p value for trend was 0.006 (Table 2).

*Association of MSI, BMI and interaction with patient characteristics*

After categorizing patients into MSS/MSI-L and MSI-H, they were further classified according to BMI into; BMI <25Kg/m^2^ and BMI ≥25Kg/m^2^. For all patients those with BMI≥25 had more than triple increased risk of MSS/MSI-L than those with normal weight (OR 3.58 (1.24-10.31); p=0.014). This significant relationship was tested for effect modification by different patient characteristics. For age, the significant association of high BMI with MSS/MSI-L was only shown in patients younger than 40 years (OR=14.9 (1.47-142.9)) and not for older patients, (OR 1.84 (0.49-6.8), p for interaction was=0.10). For gender, the association of BMI and MSI was significantly evident among females, as obese were more likely MSS/MSI-L (OR=8.06 (1.44-45.5)) but not among males (OR= 1.83 (0.44-7.63)), p for interaction = 0.187. When the relation was tested among ever and never smokers, the association of BMI with MSI was only evident among never smokers with a borderline significant interaction, p = 0.078. Regarding pathologic characteristic of the tumor, the relationship of BMI with MSI was significant only in stage III but not in early stage (I+II), for those with non-mucin secreting adenocarcinoma with non-significant p value for interaction (0.198 and 0.194 respectively). In patients with low physical activity (<600 MET minutes / week) association of BMI with MSI was not shown (OR=0.41 (0.04-3.7) but evidence of increased risk of MSS/MSI-L in obese patients was evident in group with moderate to high physical activity (OR=20 (2.2-166.6), p for interaction = 0.006 (Table 3)

**Table 1:** Relation of MSI to Clinicopathological Characteristics of 100 CRC Patients

Variables			MSI				*P*-value
			MSS/MSI-L		MSI-H		
		Total=100	N =64	%	N =36	%	
Age	<=40	28	16	57.1	12	42.9	0.373
	>40	72	48	66.7	24	33.3	
Sex	Male	44	27	61.4	17	38.6	0.626
	Female	56	37	66.1	19	33.9	
School Education/years	Illiterate	32	21	65.6	11	34.4	0.492
	<=9	24	14	58.3	10	41.7	
	10-12	32	19	59.4	13	40.6	
	>=13	12	10	83.3	2	16.7	
HPN	No	80	51	63.8	29	36.3	0.917
	Yes	20	13	65.0	7	35.0	
DM	No	86	57	66.3	29	33.7	0.368
	Yes	14	7	50.0	7	50.0	
Tumor site	Rt sided colon	23	5	21.7	18	78.3	<0.001
	Left sided colon	42	33	78.6	9	21.4	
	Rectum	35	26	74.3	9	25.7	
Tumor pathology	Non mucin Secreting Adenocarcinoma	76	51	67.1	25	32.9	0.33
Tumor	Mucin Secreting Adenocarcinoma	24	13	54.2	11	45.8	
Grade (n=91)	Low	81	52	64.2	29	35.8	0.492
	High	10	5	50.0	5	50.0	
Lymph node infiltration (n=98)	No	58	38	65.5	20	34.5	0.831
	Yes	40	25	62.5	15	37.5	
TNM staging (n=98)	I	10	6	60.0	4	40.0	0.866
	II	48	32	66.7	16	33.3	
	III	40	25	62.5	15	37.5	

MSI, Microsatellite instability; MSS/MSI-L, Microsatellite stable / Microsatellite instability low; MSI-H, Microsatellite instability High; HPN, Hypertension; DM, Diabetes Mellitus

**Table 2:** Relation of MSI to Life Style in 100 CRC Patients

Variables			MSI				*P*-value
			MSS/MSI-L		MSI-H		
		Total=100	N	%	N	%	
Smoking status	Never smoker	71	45	63.4	26	36.6	0.84
	Ever Smoker (current &former)	29	19	65.5	10	34.5	
History of alcohol consumption	No	97	63	64.9	34	35.1	0.294
	Yes	3	1	33.3	2	66.7	
Contraceptive pills (n=56)	No	36	22	61.1	14	38.9	0.571
	Yes	20	14	70.0	6	30.0	
Median consumption of fruits and vegetables (Serving/day)		100	0.71 (0.07-2.50)		0.64 (0.29-2.43)		0.837
Eating fast food (Habitual)	No	75	51	68.0	24	32.0	0.229
	Yes	25	13	52.0	12	48.0	
Eating processed meat	No	59	37	62.7	22	37.3	0.833
	Yes	41	27	65.9	14	34.1	
Physical activity	Low	59	43	72.9	16	27.1	0.026
	Moderate or high	41	21	51.2	20	48.8	
BMI	<=25	18	7	38.9	11	61.1	0.019
	25-30	33	20	60.6	13	39.4	
	>30	49	37	75.5	12	24.5	
*P-value for trend							0.006

MSI, Microsatellite instability; MSS/MSI-L, Microsatellite stable / Microsatellite instability low; MSI-H, Microsatellite instability High; BMI, Body mass index

**Table 3:** Association of BMI with MSS/MSI-L and MSI-H Colorectal Cancer and Interaction with Patient Characteristics

	MSS/MSI-L N=64		MSI-H N=36		OR (95% CI)*	*P*-value within subgroup
	BMI <25	BMI≥25	BMI <25	BMI≥25		
	N=7	N=57	N=11	N=25		
	N (%)	N (%)	N (%)	N (%)		
Age						
Age<40	1 (14.3)	15 (71.4)	6 (85.7)	6 (28.6)	14.93 (1.47 –142.9)	0.023
Age≥40	6 (54.5)	42(68.9)	5 (45.5)	19(31.1)	1.84 (0.49 – 6.80)	0.488
*P*-value for interaction				0.1		
Sex						
Males	5 (50.0)	22 (64.7)	5 (50.0)	12 (35.3)	1.83 (0.44 -7.63)	0.401
Females	2 (25.0)	35 (72.9)	6 (75.0)	13(27.1)	8.06 (1.44 -45.5)	0.014
*P*-value for interaction				0.187		
Smoking						
Never smoking	2 (20.0)	43 (70.5)	8 (80.0)	18 (29.0)	9.5 (1.85 – 50.0)	0.004
Ever smoking	5 (62.5)	14 (66.7)	3 (37.5)	7 (33.3)	1.20 (0.22 – 6.54)	1
*P*-value for interaction				0.078		
Stage						
Stage I&II	6 (50.0)	32 (69.6)	6 (50.0)	14 (30.4)	2.28 (0.63 – 8.33)	0.307
Stage III	1 (16.7)	24 (70.6)	5 (83.3)	10 (29.4)	12.0 (1.24 – 111.1)	0.021
*P*-value for interaction				0.198		
Pathology						
Non mucin secreting adenocarcinoma	4 (33.3)	47 (73.4)	8 (66.7)	17 (26.6)	5.52 (1.47-20.83)	0.007
Mucin and signet ring adenocarcinoma	3 (50.0)	10 (55.6)	3 (50.0)	8 (44.4)	1.25 (0.196-7.93)	0.813
*P*-value for interaction				0.194		
Site						
Rt side	0 (0)	5 (27.8)	5 (100.0)	13 (72.2)	1	
Lt side	3 (50)	30 (83.3)	3 (50)	6 (16.7)	5.0(0.80-31.25)	0.101
Rectum	4 (57.1)	22 (78.6)	3 (42.9)	6 (21.4)	2.75(0.48-15.87)	0.34
*P*-value for interaction				0.685		
Physical activity						
Low	6 (85.7)	37 (71.2)	1 (14.3)	15 (28.8)	0.411 (0.045-3.71)	0.661
Moderate and high	1 (9.1)	20 (66.7)	10 (90.9)	10 (33.3)	20.00 (2.23-166.6)	0.001
*P*-value for interaction				0.006		

MSS/MSI-L, Microsatellite stable / Microsatellite instability low; MSI-H, Microsatellite instability High; *odds of MSS/MSI-L with high BMI within each strata or subgroups

## Discussion

Though CRC is not one of the five most common cancers in Egypt, yet its presentation and prognosis are different from countries where it is one of the three common cancers. Age of incidence is at least one to two decades earlier than that in Western countries (Zeeneldin et al., 2012). This can be due to differences in population structures and in life expectancies. Rockhill and Giovannucci (1999) reported that Westernization of Egypt is occurring and affecting young people, as they are more likely to change their lifestyle compared to older people.

Because it is a form of heterogeneous cancer, there is interplay of both genetic and environmental factors (Nojadeh et al., 2018). In this study, we aimed at probing the association between life style factors and MSI status in CRC, which could provide more insights on its carcinogenic process.

*MSI and relation to patient characteristics and life-style*

Results of the present study showed a higher prevalence of MSI-H group (36.0%) compared to 15-20% MSI display in other studies (Vilar and Gruber et al., 2010). An Egyptian study by Nour El Hoda et al., (2017) showed a comparable percentage of MSI-H (30.8%). Previous study comparing Egyptian CRC patients with Western CRC patients, found that the frequency of MSI-H tumors in the Egyptian group was higher than that in the Western sporadic group (37% vs. 13%, p = 0.002) (Soliman et al., 2001). Soliman et al., (2019) found that the expression level of MSI-H was 47.0%, which was more than 10% higher than the present results. However, many Egyptian studies on MSI prevalence in CRC patients showed that it was higher than that reported from western population, yet there was no clear explanation for this difference. The remarkable differences in molecular pathology as compared to Western patients could result from inherent differences in sensitivity and molecular responses to Western lifestyle in Egyptians (Rockhill and Giovannucci, 1999). Overall, it may be concluded that pathogenesis of CRC in Egypt has a unique feature, which need extensive investigations.

Regarding patients, demographics age and gender were not significantly related to MSI status. These results were parallel to that by Hoffmeister et al.,(2013) and Kang et al., (2018) who found no relation between MSI and age at diagnosis, but the latter found that proportion of women were significantly higher than men among those with MSI-H CRC than MSS group.

A significant relationship was found between MSI status and tumor site where the left colon and rectum tumors tend to be more MSS while right colon tumors which tend to be more MSI-H. Results by Huang et al., (2010) and Frey et al., (2010) were matched to the current study. Jenkins et al., (2007) and Greenson et al., (2009) found that right location was also a strong predictor of MSI-H. On the contrary, Faghani et al., (2012) found that 81.8% of total MSI-H had distal tumors. The different methods used by Faghani et al., (2012) to analyze MSI frequencies through using BAT-26 and BAT-25 markers could explain the last finding.

In the present study, high-grade tumors tend to be more MSI-H without significance. Several studies found that poorly differentiated or undifferentiated histology is a strong predictor of MSI-H (Yearsley et al., 2006; Greenson et al., 2009; Frey et al., 2010; Jenkins et al., 2010; Nour El Hoda et al., 2017). On the contrary, Joel et al. (2003) found that the presence of well-differentiated tumors were important markers for microsatellite instability. Huang et al., (2010) found that MSI-H tumors were more likely to show less local aggressiveness and lower differentiation.

A significant association between smoking cigarettes and colon cancer risk have been reported in only few studies, with associations being null or weak and risk estimates of around 1.3– 1.4 (Nishihara et al., 2015; Jass et al., 2007; Guastadisegni et al., 2010; Chen et al.,2015). Studies that found a statistically significant association between cigarette smoking and colon cancer incidence indicate that dose, or amount usually smoked per day (Jass et al., 2007), is an important predictor of risk. Other studies (Moher et al., 2009; Stroup et al., 2000) that supported the dose response relationship showed stronger associations, with a relative risk of approximately 2.0, for individuals who reported smoking for 45 years or more. Current study did not find any relationship between smoking as ever and never smoking and MSI status. This could be explained by lower prevalence of ever smoker (29.0%) and current smokers (11.0%). Also 100% of females were never smoker and they constituted 56.0% of the study group. History of alcohol consumption was not found to be related to MSI as only three patients have history of alcoholic intake. Eight (three cohort and five case–control) studies investigated the association between alcohol intake and CRC according to MSI status. Overall, most studies did not find a significant association between increased alcohol consumption and MSI-H or MSS CRC (Carr et al., 2018).

In this study, neither consumption of diet with high fiber content (vegetable and fruits) nor fast food diet or processed meat were found to be associated with MSI status. A meta-analysis of case–case analyses in three studies revealed no significant difference in the association of dietary fiber intake and MSI-H colon cancer compared with MSS/MSI-L colon cancer (Carr et al., 2018).

We found that patients with low physical activity (< 600 MET minutes /week) were more likely to be MSS/MSI-L, (OR=2.56; 95% CI = 1.106 to 5.925) p value=0.026. Slattery et al., (2000) in the case-control part of study, investigated the association between physical activity and colon cancer by MSI status in both males and females. For males, intermediate and low levels of physical activity were risk factors for both MSI-H and MSS CRC. Among females, low levels of physical activity were associated with an increased risk of MSS/MSI-L colon cancer but not for MSI-H colon cancer. This relationship was not found in the case–case part of their study. Relation between low physical activity and MSS could be explained by the fact that low physically active persons tend to have higher BMI that increases risk of MSS (Campbell et al., 2007).

High BMI is an established risk factor for colorectal cancer; current study showed that the higher the BMI the more likely MSS/MSI-L tumors, p value for trend 0.006. This was supported by Campbell et al., (2007), who found that BMI, per 5 kg/m^2^, was positively associated with the risk of MSS (OR = 1.38; 95 CI = 1.24 to 1.54) and MSL (OR = 1.33; 95 CI = 1.04 to 1.72) colorectal tumors. Satia et al., (2005) found that high BMI before diagnosis was associated with the risk of MSL or MSS tumors. These results are generally consistent with those of Campbell et al., (2007), providing further evidence for the specific relationship between obesity and the risk of MSS cancer. The specificity of this link not only reinforces a causal interpretation but also points the way to a specific mechanism. 

*Association of MSI, BMI and interaction with patient characteristics*

Interaction mean that two or more factors modifies the effect of each other with regard to an outcome (Szklo and Nieto, 2019). The highly significant relation between BMI & MSI status was tested for effect modification according to patient characteristics, lifestyle factors and for clinico pathologic prognostic factors.

In this study, we found a strongly elevated risk of MSS/MSI-L CRC associated with high (≥25.0Kg/m^2^) BMI. This association was limited to younger age (<40 years old), females, never smoking, stage III, non-mucin secreting adenocarcinoma and moderate to high physically active patients.

For age, evidence of association of high BMI and MSS/MSI-L was found in young patients (<40 years). Where patients with BMI (≥25.0Kg/m2) and <40 years were more susceptible for MSS/MSI-L (OR=14.9 (1.47-142.9), p=0.02) while odds for age ≥ 40 years was (1.8(0.49-6.8), p=0.488) and p for interaction =0.10. This result could be explained by Westernization of Egypt affecting younger people, as they are more likely to change their lifestyle compared to older people (Rockhill and Giovannucci, 1999). Parallel with our results, Campbell et al., (2007) found that high BMI at age of 20 years, modeled as a continuous variable in 5Kg/m^2^ increment, was positively associated with risk of MSI/L but not MSS or MSI-H. This difference could be explained by difference in MSI definitions in this study as we used two levels of MSI (high versus stable and low), whereas Campbell et al., (2007) characterized three level (MSS, MSL and MSI-H). On the contrary, Hoffmeister et al., (2013), (in the case only part of their study) found that the relation between BMI and MSI was not modified by age categorized as less than70 and ≥70 years (OR=1.37 (0.97-1.92), 1.51 (1.07-2.12) respectively, p for interaction=0.59. Difference in results between present study and their study could be explained by different categorization of age. 

In the present study, the association of BMI and MSI was shown to be affected by gender, where females odds of being MSS/MSI-L with high BMI was 8.06 (1.44-45.5) compared to those with low BMI, p=0.014. For males this relationship was not evident (OR=1.83(0.44-7.63), p for interaction =0.187. Despite the fact that we had small number of both males and females with low BMI, which influenced p value for interaction, yet the relation indicates risk evidence for females to have MSS/MSI-L when they are obese. Complex interplay between hormonal factors, fat distribution and tumor biology could be underlying these sex differences to be further elucidated.

Likewise, a Swedish cohort study found higher risk of MSS CRC for women with high BMI but not for males (Brandstedt et al., 2013). Multivariable analysis of the US Health Professionals follow-up study and the Nurses’ health study found increasing risk of MSS CRC with increasing BMI (Carr et al., 2018). On the contrary, Hoffmeister et al., (2013) found that per 5 kg/m^2^ BMI the risk of MSI-H for females increased by 84.0% (OR=1.84(1.34–2.52)) with no evidence among males p for interaction = 0.02. An explanation for this difference is that a good proportion of CRC cases in their study was not sporadic but hereditary. Another study found that relationship between BMI and MSS/MSI-L was modified by gender but was shown only among males not females (Slattery et al., 2000). Campbell et al., (2007) did not find an interaction between BMI, MSI and gender p for interaction =0.22.

The association of BMI with MSI was only evident among never smokers with a borderline significant interaction, p = 0.078. This result could be justified by the fact that more than half of the study group were females who were never smokers, and only 29.0% of patients were ever smokers.

For pathologic characteristic of the tumor, the relationship of BMI with MSI was significant only in stage III but not in early stage (I+II) and for those with non-mucin secreting adenocarcinoma with non-significant p value for interaction (0.198 and 0.194 respectively). Levi et al., (2011) showed that tumor histology of mucinous cancer is highly correlated with MSI-high tumor and serves as surrogate marker for MSI-high tumors. They reported that the increased risk of MSS associated with increased BMI was evident only for the non-mucinous cancer; they reported that their results were consistent with those of Campbell et al., (2007). Current results also found that non-mucin secreting tumors are risk for MSS/MSI-L in patients with high BMI which are analogous to Campbell et al., (2007) and Levi et al., (2011) results.

In patients with low physical activity (<600MET minutes/ week) association of BMI with MSI was not shown (OR=0.41(0.04-3.7)) but evidence of increased risk of MSS/MSI-L in obese patients was evident in-group with moderate to high physical activity (OR=20.0 (2.2-166.6), p for interaction = 0.006. One case–control study (Slattery et al., 2000) investigated the association between physical activity and colon cancer by MSI status and studied the effect of gender on the relation between physical activity and MSI across strata without testing for interaction. In the case control part, they found that for males, intermediate and low levels of physical activity were risk factors for both MSI-H and MSS CRC. Among females, low levels of physical activity were associated with an increased risk of MSS/MSI-L colon cancer but no association was observed for MSI-H colon cancer. In a case–case analysis comparing MSI-H with MSS colon cancer, no difference was identified. Larsson et al., (2006) evaluated the possibility of the interaction between physical activity and BMI in relation to CRC risk. They classified participants according to both vigorous leisure-time physical activity and BMI and found that the decreased risk of colorectal cancer associated with increased levels of leisure-time physical activity was observed across all categories of BMI (p for interaction= 0.33).

In summary, high BMI was strongly associated with MSS/MSI-L CRC, which are known to be bad prognostic subgroups. Though effect modification mostly was not found significant, limited by the relatively small number of subgroups, this relation was getting ready to be influenced by some prognostic factors including, tumor site, TNM stage, tumor pathology and a number of patient’s characteristics and life style factors including age, gender and physical activity. 

The underlying basis for the increased risk of MSS tumors associated with overweight or obesity and the lack of an association with MSI-H tumors remain largely speculative. However, one possible explanation includes the matrix metalloproteinase system, which is involved in diet-induced obesity through remodeling of the extracellular matrix that surrounds the expanding adipose tissue (Halberg et al., 2008) and in the degradation of the extracellular matrix during colorectal cancer metastasis (Rydlova et al., 2008).

Another potential biological source of increased risk of MS-stable tumors from overweight and obesity not with MSI-H tumors involves telomeres (the physical ends of chromosomes): An inverse association between telomere length and body weight was reported (Kim et al., 2009), and shorter telomere length, in turn, has been linked to chromosomal instability and MS-stable colorectal tumors but not to MSI-high colorectal tumors (Lengauer et al., 1997). Additional studies will be needed to better understand the differing etiologies of MSS/MSI-L and MSI-H colorectal cancers.

Despite the limitations faced, one of the advantages of this study is that, it is the first molecular pathologic epidemiology study probing the effect modification of patient characteristics on the association between BMI and MSI gene in Egyptian patients with extensive data collection. On the other hand, study was limited to 100 patients due to restricted resources and economic difficulties in Egypt, which was reflected on subgroup analyses and multiplicity of comparisons with increased chance of random errors. In addition to a limitation of using IHC that may lead to reaction bias or interpretation bias

In conclusion, this study confirms previous findings of increased risk of MSS/MSI-L colorectal cancer with obesity and suggests that obesity may also be associated with increased risk of MSS/MSI-L colorectal cancer among women, younger patients, late stage, non-mucin secreting adenocarcinoma and physical activity. Further research is needed to confirm present results
